# “Paradoxical” findings of tumor vascularity and oxygenation in recurrent glioblastomas refractory to bevacizumab

**DOI:** 10.18632/oncotarget.21978

**Published:** 2017-10-24

**Authors:** Yohei Yamamoto, Ryota Tamura, Toshihide Tanaka, Kentaro Ohara, Yukina Tokuda, Keisuke Miyake, Jun Takei, Yasuharu Akasaki, Kazunari Yoshida, Yuichi Murayama, Hikaru Sasaki

**Affiliations:** ^1^ Department of Neurosurgery, Jikei University School of Medicine Kashiwa Hospital, Kashiwa-shi, Chiba 277-8567, Japan; ^2^ Department of Neurosurgery, Keio University School of Medicine, Shinjuku-ku, Tokyo 160-8582, Japan; ^3^ Division of Diagnostic Pathology, Keio University School of Medicine, Shinjuku-ku, Tokyo 160-8582, Japan; ^4^ Department of Neurosurgery, Kagawa University Hospital, Kita-gun, Kagawa 761-0793, Japan; ^5^ Department of Neurosurgery, Jikei University School of Medicine, Minato-ku, Tokyo 105-8461, Japan

**Keywords:** bevacizumab, glioblastoma, vascular endothelial growth factor, hypoxia, neoadjuvant therapy

## Abstract

Anti-angiogenic therapy induces the apparent normalization of vascular structure, decreases microvessel density (MVD), and improves tumor oxygenation in glioblastomas (GBMs). Six initial and recurrent tumor pairs after bevacizumab (Bev) treatment were compared with GBMs from nine patients resected under neoadjuvant Bev treatment with regard to histological characteristics; MVD; MIB-1 index; and expression of vascular endothelial growth factor (VEGF) and its receptors, hypoxia markers (hypoxia-inducible factor 1 alpha, carbonic anhydrase 9), and nestin as a marker of glioma stem-like cells. In recurrent tumors post-Bev treatment, while the MVD remained low compared with the paired initial tumors (pre-Bev tumors), the expression of hypoxic markers were increased and were even higher in expression compared with the paired pre-Bev tumors in three of the six cases. MIB-1 indices were similar among the initial GBMs, neoadjuvant group, and recurrent tumors post-Bev treatment. The nestin-positive cell ratio of the post-Bev recurrent tumors was as high as that of the pre-Bev tumors. The expression of VEGF and VEGFR1 was increased in the post-Bev recurrent tumors in three and four cases, respectively, compared with the paired pre-Bev tumors. In the majority of Bev-refractory GBMs, tumor hypoxia was present with a paradoxical decrease in MVD. These findings suggest that re-activation of tumor angiogenesis is not initially involved in the acquisition of resistance to Bev.

## INTRODUCTION

Glioblastoma (GBM) is a highly vascularized malignant tumor, and thus, inhibiting angiogenesis is an attractive treatment strategy. Vascular endothelial growth factor (VEGF) promotes endothelial cell proliferation and migration, and consequently, tumor angiogenesis and tumor growth. VEGF expression correlates with prognosis in glioma patients and histological grade of malignancy [1].

Bevacizumab (Bev) is a recombinant, humanized monoclonal antibody that inhibits VEGF-A. Data from randomized clinical trials have suggested that Bev administration is associated with favorable event-free survival and improvement in patients with a poor performance status [2–4], and it is increasingly being used for the treatment of newly diagnosed and recurrent high-grade gliomas in Japan. However, the response of GBM to Bev is invariably transient, and recurrent tumors after Bev treatment are associated with a more aggressive and invasive phenotype [5–7]. Because the targets of Bev are not tumor cells but are vascular endothelial cells, the mechanism of resistance to Bev is likely different from those of other chemotherapeutic agents that target tumor cells. However, the mechanisms of resistance/refractoriness as well as the response to Bev treatment have not been fully elucidated [8]. *In situ* observations of surgical specimens are likely to be crucial for clarifying these issues. We previously demonstrated that Bev induces the apparent normalization of vascular structure, decreases microvessel density (MVD), and improves tumor oxygenation in human GBMs resected under Bev treatment [9].

In this study, we used histopathological specimens of initial and recurrent tumor pairs after Bev treatment and GBMs resected following neoadjuvant Bev treatment to investigate the molecular/histopathological differences in tumors under Bev treatment compared with refractory tumors, as well as those of initial tumors versus refractory tumors. To the best of our knowledge, this is the first report of *in situ* observations showing the difference between tumors that responded and those that acquired resistance to anti-angiogenic therapy.

## RESULTS

### Clinical characteristics of the patients

The clinical course of the nine patients in the neoadjuvant group is summarized in Table 1. Specifically, the Karnofsky performance status of these patients dramatically improved from 50–80 to 80–100 with one to three doses of pre-operative Bev. A median sum of perpendicular diameter (SPD) change in tumor volume induced by Bev treatment was –43% (–8% to –61%). Seven newly diagnosed patients were all alive as of April 2017, and overall survival ranged from 10 to 26 months following the first administration of Bev. The clinical characteristics of the six patients from whom paired pre-Bev and post-Bev tumors were obtained are summarized in Table 2. Three of the six patients underwent a second surgery at the time of recurrence following Bev treatment, and the remaining three patients were deceased at the time of recurrence. The tumors from the latter three patients were post-mortem. Regarding the radiological progression patterns of the post-Bev recurrent tumors, cases 10 and 11 had contrast-enhancement at progression (cT1 flare-up), whereas cases 12–15 had non-enhanced tumors (case 12: T2 circumscribed; cases 13–15: T2 diffuse). The interval between final Bev treatment and post-Bev resection or death ranged from 31 to 208 days.

**Table 1:** Neoadjuvant Bev group

Case	Age	Sex	Location	Histological diagnosis under neoadjuvant bev	IDH-1 status	Clinical stage	KPS before bev	Treatment before surgery	KPS before surgery (after neoadjuvant Bev)	Interval between last Bev to surgery	T1 Gd.
1	55	M	Rt. frontal	High-grade glioma	WT	newly diagnosed	50	bev 1 course	80	21 days	–61%
2	77	F	Lt. parietal	High-grade glioma	WT	newly diagnosed	70	bev 1 course	80	26 days	–14%
3	83	M	Lt. frontal	High-grade glioma	WT	newly diagnosed	70	bev 1 course	100	27 days	–43%
4	68	M	Rt. occipital	High-grade glioma	WT	newly diagnosed	90	bev 1 course	100	21 days	–38%
5	53	F	Rt. frontal	High-grade glioma	WT	newly diagnosed	50	bev 2 course	90	21 days	–57%
6	72	M	Lt. frontal	High-grade glioma	WT	newly diagnosed	40	bev 1 course	60	28 days	–20%
7	80	F	Lt. insular	High-grade glioma	WT	newly diagnosed	50	bev 1 course	80	21 days	–24%
8	49	M	Rt. frontal	High-grade glioma	WT	newly diagnosed	80	tmz 1 course & bev 2 courses	90	21 days	–52%
9	48	M	Lt. temporal	GBM	WT	recurrent	80	bev 3 courses	80	36 days	–8%

**Table 2:** Paired samples of pre- and post-Bev

Case	Age	Sex	Location	Initial histological diagnosis	IDH-1 status	Clinical course and adjuvant therapy between initial and 2nd surgery	KPS before bev	Pattern of recurrence after bev^*^	KPS before 2nd surgery/autopsy	Interval between last bev to 2nd. surgery/autopsy
10	48	M	Rt. parietal	GBM	WT	RT, TMZ (14 cycles) , Bev (16 cycles)	60	cT1 flare-up	80	33 days
11	50	F	Lt. frontal	GBM	WT	RT, TMZ ( 7 cycles), Bev (10 cycles)	70	cT1 flare-up	70	33 days
12	65	M	Rt. parietal	GBM	WT	RT, TMZ (17 cycles), Bev (19 cycles)	50	T2 circumscribed	50	31 days
13	52	F	Lt. temporal	GBM	WT	RT, TMZ ( 9 cycles) , Bev (16 cycles)	70	T2 diffuse	10	35 days
14	73	M	Lt. temporal	GBM	WT	RT, TMZ (13 cycles), Bev (26 cycles)	50	T2 diffuse	10	35 days
15	41	M	Lt. cerebellar	GBM	WT	RT, TMZ (26 cycles), Bev (26 cycles)	100	T2 diffuse	10	208 days

### Histological findings

### Comparison of MVD and proliferative activity among neoadjuvant, pre-Bev, and post-Bev recurrent groups

#### Tumors resected under Bev treatment (neoadjuvant group)

**Figure 1:**
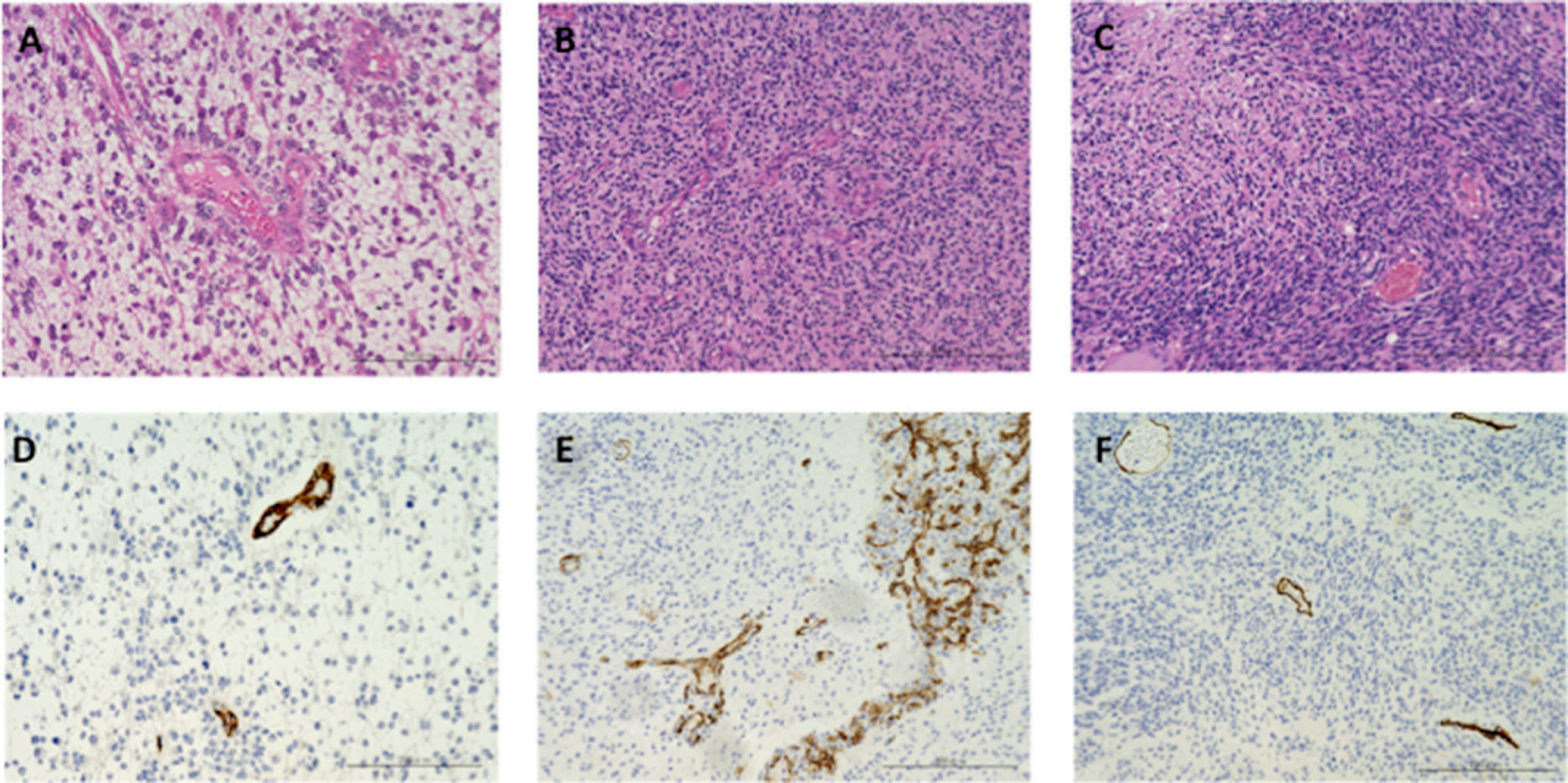
Photomicrograph of GBM resected under neoadjuvant Bev and paired initial GBM and recurrent GBM after Bev therapy (**A**–**C**) hematoxylin and eosin staining. (**D**–**F**) immunohistochemical analysis of CD34. A and D: tumor resected under neoadjuvant Bev in case 2 (magnification: 200x, magnification bar: 200 μm). Note that the tumor cells are predominantly accumulated around the vessels, and the interstitial cell density is relatively sparse. MVD is clearly decreased (17.6/5 HPF), and microvascular proliferation is not observed. B and E: initial tumor in case 10 (magnification: 200×, magnification bar: 200 μm). Typical glomeruloid vessels are observed. C and F: recurrent tumor after Bev in case 8 (magnification: 200×, magnification bar: 200 μm). Note that MVD is still low (17.6/5 HPF) with paradoxical high cellularity.

Tumor cells showed pleomorphic cells with nuclear atypia and mitotic activity, but typical microvascular proliferation and palisading necrosis were inconspicuous, and “glomeruloid” microvasculature was occasionally collapsed in cases 1–9. Immunohistochemistry for R132H-mutant isocitrate dehydrogenase (IDH)-1 were negative in all nine tumors. Histopathological diagnosis was high-grade glioma, equivalent to grade III or grade IV. The tumor in case 9, the minimal responder to Bev (–8%; Table 1), was mainly composed of medium-sized astrocytic cells with increased cellularity and abundant mitotic figures. Palisading necrosis was occasionally observed and microvascular proliferation was absent. The histopathological diagnosis was GBM. The mean MVD was 24.0/5 high-power field (HPF) (12.8 to 62.6), and the mean MIB-1 index was 21.3%. These data are shown in Figure 1A and 1D.

### Pre-Bev initial tumors

The pre-Bev initial tumors had the typical appearance of GBMs with increased cellularity and abundant mitotic figures. Palisading necrosis and microvascular proliferation were present, and immunohistochemistry for R132H-mutant IDH-1 were negative in all six tumors. The histopathological diagnosis was GBM. The mean MVD was 69.9/5 HPF (13.2 to 113), and the mean MIB-1 index was 23.3%. These data are shown in Figure 1B and 1E.

### Post-Bev recurrent tumors

The recurrent tumors after Bev treatment were characterized by microbleeding, which was significant in two of the six tumors, and there was less microvasculature compared with the paired initial tumors. Palisading necrosis was observed in four tumors. The mean MVD was 26.8/5 HPF (17.6 to 47.2), and the mean MIB-1 index was 18%. The MVD of the post-Bev recurrent tumors was higher than that in the neoadjuvant group; however, it was still significantly lower than the paired initial tumors (mean MVD: neoadjuvant, 24.0; initial, 69.9; post-Bev recurrent, 26.8; *p =* 0.00871 by one-way ANOVA followed by post-hoc test; *p <* 0.05 for neoadjuvant vs. initial, not significant (NS) for neoadjuvant vs. post-Bev recurrent, *p <* 0.05 for initial vs. post-Bev recurrent). In contrast, there was no significant difference in the MIB-1 proliferative indices among the initial GBM group, neoadjuvant group, and post-Bev recurrent group (*p =* 0.894 by one-way ANOVA followed by post-hoc test; NS for neoadjuvant vs. initial, NS for neoadjuvant vs. post-Bev recurrent, NS for initial vs. post-Bev recurrent). These data are shown in Figure 1C, 1F and Table 4.

**Table 3:** Summary data for immunohistochemistry

	Case	Age	Sex	MVD by CD34 staining	MIB-1 index (%)	VEGF-A	VEGFR1	VEGFR2	HIF-1α	CA-9	Nestin-positive cell ratio (%)
neoadjuvant group	1	55	M	**19.4**	**10.9**	**-**	**-**	**+**	**-**	**-**	**6.7**
	2	77	F	**15.4**	**20**	**++**	**+**	**+**	**-**	**+**	**29.1**
	3	83	M	**17.6**	**70**	**+**	**+**	**+**	**-**	**+**	**39.4**
	4	68	M	**62.6**	**10**	**+**	**+**	**+**	**+**	**+**	**47**
	5	53	F	**39.8**	**20**	**-**	**+**	**+**	**+**	**+**	**25**
	6	72	M	**17.8**	**8**	**+**	**-**	**+**	**+**	**++**	**19**
	7	80	F	**16.4**	**30**	**++**	**++**	**+**	**-**	**+**	**62**
	8	49	M	**14.2**	**3**	**-**	**-**	**+**	**+**	**+**	**16.2**
	9	48	M	**12.8**	**20**	**++**	**++**	**+**	**++**	**+**	**16.6**
Initial versus recurrent/autopsied group	10–1 (pre.Bev.)	48	M	**105.8**	**20**	**+**	**+**	**+**	**++**	**+**	**90.1**
	10–2 (post.Bev.)			**17.6**	**22**	**+**	**++**	**+**	**++**	**++**	**96**
	11–1 (pre.Bev.)	50	F	**113**	**15**	**+**	**++**	**+**	**++**	**+**	**98**
	11–2 (post.Bev.)			**26.8**	**15**	**+**	**++**	**+**	**++**	**++**	**91**
	12–1 (pre.Bev.)	65	M	**13.2**	**10**	**+**	**+**	**+**	**+**	**++**	**99**
	12–2 (post.Bev.)			**29.8**	**1**	**+**	**++**	**+**	**+**	**++**	**44**
	13–1 (pre.Bev.)	52	F	**99.8**	**15**	**-**	**+**	**+**	**++**	**+**	**91**
	13–2 (post.Bev.)			**26.2**	**15**	**++**	**++**	**+**	**++**	**++**	**70.4**
	14–1 (pre.Bev.)	73	M	**64.8**	**50**	**++**	**+**	**+**	**++**	**++**	**65**
	14–2 (post.Bev.)			**47.2**	**5**	**++**	**++**	**+**	**++**	**++**	**23**
	15–1 (pre.Bev.)	41	M	**22.6**	**30**	**++**	**+**	**++**	**++**	**++**	**82**
	15–2 (post.Bev.)			**13.2**	**20**	**+**	**++**	**+**	**++**	**++**	**32**

**Table 4:** Comparison of mean of MVD, MIB-1 index, and nestin-positive cell ratio in neoadjuvant, pre-bev, and post-bev groups

	neoadjuvant group	paired samples		*p*-value
		pre-bev initial tumors	post-bev recurrent tumors	
MVD by CD34 staining (/5HPF)	24.0 ± 16.6	69.9 ± 43.6	26.8 ± 11.8	0.00871
MIB-1 index (%)	21.3 ± 20.0	23.3 ± 14.7	11.6 ± 8.5	0.874
Nestin-positive cell ratio (%)	29.0 ± 17.5	87.5 ± 12.6	59.4 ± 30.9	0.00021

### Comparative analyses of VEGF/VEGFR expression among neoadjuvant Bev, pre-Bev, and post-Bev groups

**Figure 2:**
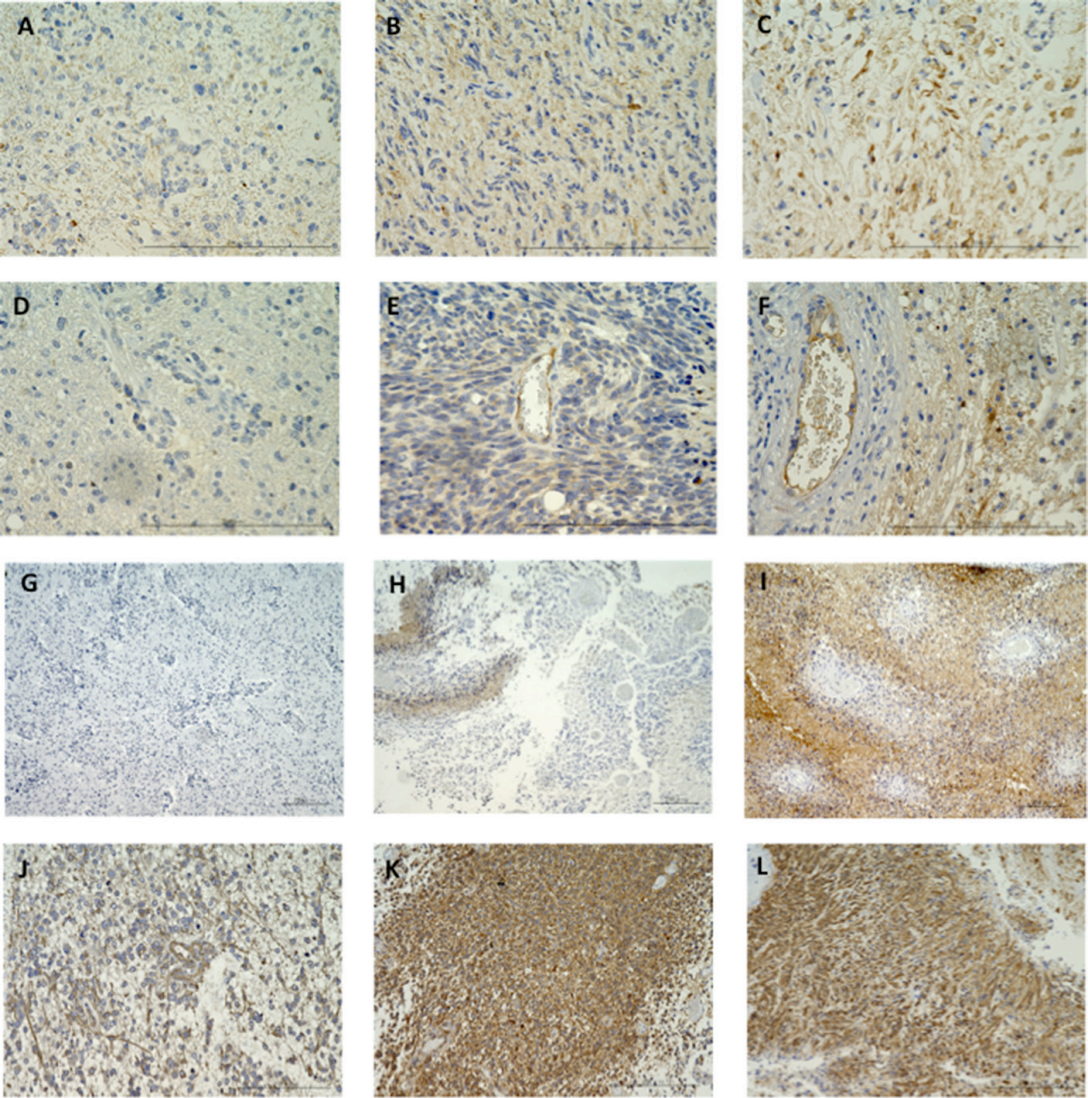
Immunohistochemistry for VEGF (**A**–**C**) and VEGFR1 (**D**–**F**) in GBMs resected under neoadjuvant Bev before and after Bev therapy. A and D: neoadjuvant Bev in case 1. Expression of VEGF and VEGFR1 was almost negative (magnification: 400×, magnification bar: 200 μm). B and E: Before Bev therapy in case 8. Moderate to weak level of expression was observed for both VEGF and VEGFR1 (+, +, respectively) (magnification: 400×, magnification bar: 200 μm). C and F: After Bev therapy in case 12. Expression of VEGF and VEGFR1 was stronger than before Bev therapy (+, ++, respectively) (magnification: 400x, magnification bar: 200 μm). Immunohistochemistry for CA9 in GBMs resected under neoadjuvant Bev, before and after Bev therapy (**G**–**I**). G: neoadjuvant Bev in case 1. Expression of CA9 is negative (magnification: 100×, magnification bar: 200 μm). H: Before Bev therapy in case 6. CA9 is expressed occasionally around necrotic regions (assessed as +) (magnification: 100×, magnification bar: 200 μm). I: After Bev therapy in case 10. Universal, strong expression of CA9 is observed (assessed as ++) (magnification: 100×, magnification bar: 200 μm). Note that CA9 expression in recurrent group was upregulated after Bev failure compared with before Bev. Immunohistochemistry for nestin in GBMs resected under neoadjuvant Bev, before and after Bev therapy (**J**–**L**). J: neoadjuvant Bev in case 2 (magnification: 200x, magnification bar: 200 μm). Nestin staining is clearly decreased, and nestin-positive cells are predominantly found around vessels. Nestin-positive cell ration is 6.7%. K: Before Bev therapy in case 10 (magnification: 200×, magnification bar: 200 μm). Nestin-positive cell ratio is 98%. L: After Bev therapy in case 10 (magnification: 200×, magnification bar: 200 μm). Nestin-positive cell ratio is 91%. Note that the positive cell ratio of nestin expression in a tumor resected under neoadjuvant Bev is clearly decreased compared with initial or Bev-refractory tumors, whereas same level of expression is observed in initial tumor and recurrent tumor after Bev.

VEGF expression was negative in the three best responders (–61% for case 1, –57% for case 5, –52% for case 8) of the nine patients in the neoadjuvant group (Figure 2A, Tables 1, 3), and was negative or very faint in four of the six patients with pre-Bev tumors (Figure 2B, Table 3). VEGF expression was clearly observed in the other 14 tumors including 6 post-Bev recurrent tumors. VEGFR1 expression was negative or faint in 5 of the 9 patients in the neoadjuvant group, but was clearly observed in the other 16 tumors (Table 3). Similarly, VEGFR2 expression was faint in 3 of the 9 cases in the neoadjuvant group, but was clearly observed in the other 18 tumors. Interestingly, in comparing the initial and post-Bev paired tumors, VEGF and VEGFR1 expression was increased in the post-Bev tumors in four and five of the six cases, respectively, including two cases that had a cT1 flare-up pattern (Figure 2C–2F, Table 3).

### Comparative analyses of hypoxia marker (HIF-1α and CA9) expression among neoadjuvant Bev, pre-Bev, and post-Bev groups

CA9 is a hypoxia-inducible enzyme as well as a useful biomarker for predicting the poor prognosis of GBM [10]. Transcriptionally active HIF-1α is bound to the hypoxia response element in the promoter area of the CA9 gene, thus inducing CA9 expression [11, 12]. Expression of HIF-1α was not observed in four of the nine cases in the neoadjuvant group, and CA9 expression was faint or negative in three of the nine tumors (Figure 2G). Thus, improved oxygenation was suggested in all but one minimal responder (–8% for case 9; Table 1).

In contrast, both HIF-1α and CA9 were clearly expressed in all of the pre-Bev initial and post-Bev recurrent tumors (Figure 2H, 2I), suggesting that tumor oxygenation was improved with Bev treatment but returned to the original hypoxic condition in tumors refractory to Bev. Interestingly, in comparing pre-Bev initial and post-Bev paired tumors, CA9 expression was increased in post-Bev recurrent tumors in three (cases 10, 11, 13) of the six cases including two of the three enhancing patterns of recurrence (Figure 2G–2I, Table 3).

### Comparative analyses of stem cell marker (nestin) expression among neoadjuvant Bev, pre-Bev, and post-Bev groups

Nestin is a stem cell marker, and is also an angiogenesis marker of proliferating vascular endothelial cells [13]. Nestin-positive cells were significantly less frequent in tumors resected under neoadjuvant Bev than in pre-Bev initial or post-Bev recurrent tumors, and there was a significant difference in the nestin-positive cell ratio between pre-Bev initial and post-Bev recurrent tumors (Figure 2J–2L, Table 3). (mean positive cell ratio: neoadjuvant 29.0%, pre-Bev initial 87.5%, post-Bev recurrent 59.4%, *p =* 0.00021 by one-way ANOVA followed by post-hoc test ; *p <* 0.001 for neoadjuvant vs. initial, *p <* 0.005 for neoadjuvant vs. post-Bev recurrent, NS for initial vs. post-Bev recurrent) CA9- and nestin-positive cells co-localized in the perivascular areas (Figure 2I–2L, Tables 3, 4).

## DISCUSSION

In this study, we compared paired initial and recurrent GBMs after Bev treatment, in addition to GBMs resected following neoadjuvant Bev treatment. In the neoadjuvant group, as previously reported [9], improvement of tumor oxygenation was noted in eight of the nine tumors, and the MVD and nestin-positive cell ratio were significantly decreased compared with the pre-Bev initial tumors. On the other hand, in the post-Bev recurrent tumors, while the MVD remained decreased compared with the paired initial tumors, the hypoxic markers were re-expressed and were even more prominent in expression compared with the paired initial tumors in three of the six cases. There was no significant difference in MIB-1 indices between the pre-Bev initial, neoadjuvant, and post-Bev recurrent groups. The nestin-positive cell ratio of the post-Bev recurrent tumors was as high as that of the initial tumors. The expression of VEGF and VEGFR1 was increased in the post-Bev recurrent tumors compared with their paired initial tumors in three and five of the six cases, respectively.

The observed decreased HIF-1α expression in the neoadjuvant group supports previous data that vessel regression induced by anti-VEGF therapy is associated with an increase in median pO_2_ and a decrease in hypoxia below 5 mmHg [14]. The improvement of tumor oxygenation could be explained by a decrease in the number of tumor and endothelial cells consuming oxygen. The *in situ* observation in pre- and post-Bev GBMs is crucial to elucidate the mechanism of action as well as resistance to Bev; however, only a few such studies have been reported to date [9, 15–17].

DeLay *et al.* [17] performed comparative analyses of the paired pre-treatment and post-Bev recurrent tumors from 21 GBM patients. The authors classified the cases into two subtypes based on radiographic characteristics of the post-Bev recurrent tumors (i.e., enhanced and non-enhanced Bev-resistant GBMs). According to their findings, the non-enhancing pattern of recurrence revealed reduced MVD, the increased expression of hypoxic markers, and unchanged proliferative activity compared with the pre-Bev paired tumors. In contrast, the enhancing pattern of recurrence was characterized by an unchanged MVD, unchanged expression of hypoxic markers, and increased proliferation compared with pre-Bev paired tumors. The expression of VEGF and VEGFR2 was similar between the paired pre- and post-Bev tumors with a non-enhancing or enhancing recurrence pattern.

However, we did not observe clear differences in the immunohistochemical findings in recurrent GBMs between enhanced and non-enhanced as described above, although the reason and mechanism remain unclear. It is possible that the heterogeneity among patients in the different treatment groups caused the differences in MVD and hypoxic marker expression in the Bev-resistant GBMs between our study and the previous one, since more than half of the cases in the previous study had various chemotherapeutic agents combined with Bev and all six patients in this study had radiotherapy and temozolomide in addition to Bev.

Nonetheless, this study, together with previous studies [9, 16, 17], confirmed that MVD is significantly decreased with improvement of tumor oxygenation in GBMs during effectiveness of Bev, and that in the majority of Bev-refractory GBMs, tumor hypoxia is regained to a similar or even higher level compared with the paired pre-Bev GBMs while the MVD is still low. HIF-1α directly regulates the expression of VEGF; therefore, the degree of tumor oxygenation and tumor vascularity are generally parallel [18, 19]. However, in post-Bev recurrent tumors, tissue oxygenation and MVD were paradoxically opposite. These findings indicate that the re-activation of tumor angiogenesis is not involved in the first stage of refractoriness to Bev.

In this study, there was a relative trend towards upregulation of VEGF and VEGFR1 in the post-Bev recurrent tumors compared with their paired initial tumors. Hypoxia in tumor tissue is caused by oxygen consumption without balance with its supply, which is the most dynamic effect on tissue pO_2_ among several factors such as irregular vascular geometry, vascular density, and altered blood viscosity [20]. Vascular density in tumor tissue usually decreases after anti-angiogenic therapy, which should cause oxygen concentrations to decrease; however instead, paradoxical oxygenation is induced [9, 14, 17, 21, 22]. During responsiveness to Bev, probably because oxygen demand by the tumor decreases, hypoxic markers (HIF-1α and CA9) and the marker for stem cell and vascular proliferation (nestin) are decreased [9], suggesting that tumor oxygenation is preserved; thus, VEGF and its receptors are relatively reduced. Whereas, when the tumor represents refractoriness to Bev, oxygen demand by the tumor increases and the microenvironment of the tumor tissue becomes hypoxic, causing stemness with chemoresistance and radioresistance. In addition, VEGF and its receptors are relatively increaesd under hypoxia, leading to inhibitory effects on tumor immunity.

It is also possible that activation of alternative angiogenic factors occurs at the time of recurrence during Bev therapy. Platelet-derived growth factor, fibroblast growth factor, ephrin, angiopoietin, and invasion-mediating genes such as integrin and matrix metalloproteinase (MMP) 2 and 9 are induced during resistance of VEGF blockade [8, 23–25]. Stromal cell-derived factor is also involved in rebuilding of vasculature at the time of tumor recurrence post-Bev treatment [8].

The salvage angiogenic signaling pathway involving KRAS and NF-κB was activated via upregulation of interleukin-8 [26]. VEGF/HIF-1α/mammalian target of rapamycin (mTOR) signaling pathway could be related to the change of tumor metabolism; thus expression of mTOR might be altered by Bev therapy [27]. In addition, the bone marrow-derived mononuclear cells, CD11b-positive myeloid cells [28, 29], and Tie2-expressing monocytes [30] were induced at the time of refractoriness to anti-VEGF therapy, accompanied with production of MMP 2 and 9 under hypoxic condition resulting in invasive change. These molecules/pathways might indeed be involved in the initial mechanism of resistance, and among the therapeutic targets to prevent the acquisition of resistance to Bev. With respect to involvement of an alternative angiogenic pathway following Bev failure, various molecules and signal pathways as described above should be investigated in the future.

The major limitation of this study was the small sample size due to the rarity of cases that underwent surgery following tumor progression after Bev therapy. Moreover, the tumors resected under neoadjuvant Bev and the Bev-refractory tumors were not from the same patients. Future studies will ideally use pairs of tumors resected under neoadjuvant Bev and post-Bev refractory tumors to confirm our results.

In conclusion, the results of this study together with previous studies demonstrated that in GBMs under Bev treatment, MVD is significantly decreased with improvement of tumor oxygenation, and that in the majority of Bev-refractory GBMs, tumor hypoxia is recovered with a paradoxical decrease in MVD. These *in situ* comparative analyses of samples from the same patients pre- and post-Bev treatment will help to understand the mechanism of resistance to anti-angiogenic therapy and to develop optimal therapy that can be used in the clinic.

## MATERIALS AND METHODS

### Patients and tissues

This study used 21 glioblastoma tissues from 15 patients obtained at three different settings: nine tumors resected under neoadjuvant Bev (defined as the neoadjuvant group, *n* = 9) and paired tumors of pre- and post-Bev treatment from another six patients (defined as pre-Bev initial group and post-Bev recurrent group, respectively). The nine patients in the neoadjuvant group (cases 1–9) had been treated with one to three courses of pre-operative Bev at a dose of 10 mg/kg, and resection was performed 21–36 days after the last administration of Bev (Table 1). Eight of the nine patients (cases 1–8) were newly diagnosed cases. One patient (case 9) was treated with Bev at tumor recurrence, and resection was performed under Bev treatment.

From another six patients, the paired pre-Bev initial and post-Bev recurrent tumors were available. Four of the six patients (cases 10, 11, 12, and 14) were treated with the Stupp regimen (radiotherapy (60 Gy, 30 fractions) concomitant with temozolomide at a dose of 75 mg/m^2^), temozolomide (150 mg/m^2^), and Bev following initial resection, and two patients (cases 13 and 15) were treated with Bev at the tumor recurrence following Stupp regimen and temozolomide (150 mg/m^2^). All six recurrent tumors were refractory to Bev despite initial response to Bev; three were resected tumors following progression after Bev and three were postmortem (Table 2). Bev was administered at a dose of 10 mg/kg every two weeks.

All 21 tumors were examined for histological features, MVD, VEGF/VEGFR, HIF-1α/CA9 as markers of hypoxia in the tumor tissue, and nestin as a marker for glioma stem-like cell population. The 21 tumors were obtained at Jikei University Kashiwa Hospital, Kagawa University Hospital, and Keio University Hospital. Laboratory experiments were performed at Keio University School of Medicine. This translational research was approved by the Institutional Review Board at each of three institutes.

### Radiological assessment

Tumor response to Bev was assessed by the SPD [31]. Patterns of recurrence were classified as cT1 flare-up (two patients), T2 diffuse (one patient), or T2-circumscribed (two patients) as follows [32]. (1) cT1 flare-up: characterized by an initial decrease in contrast enhancement (CE) on T1-weighted images after treatment initiation, and an increase (flare-up) of CE again at tumor progression. T2 signal stays stable or increased. (2) T2 diffuse: characterized by a signal increase on T2-weighted images with a poorly defined border despite the fact that CE on T1-weighted images remains decreased. Hypointensity on T1-weighted images is faint and disproportionally smaller than T2 hyperintensity. (3) T2 circumscribed: characterized by a signal increase on T2-weighted images with a bulky structure and sharp borders that correspond to a T1 hypointense signal. CE on T1-weighted images remains decreased, or only a few faintly speckled CE lesions are visible.

### Immunohistochemical analyses

Formalin-fixed paraffin embedded sections (thickness of 4 μm) were stained using an immunoperoxidase technique as previously described [9]. IDH-1 status was assessed as R132H IDH-1 immunohistochemistry (1:50, H09, Dianova, Hamberg, Germany). Tumor proliferative potential was evaluated as MIB-1 positivity as previously described (DAKO, Glostrop, Denmark) [33]. MVD was assessed using CD34 immunohistochemistry (1:100, QBEnd10, DAKO) at lower power field (40x), and the five most vascularized areas (hot spots) were selected. Microvessel counting was performed on five of these areas at HPF (200x, 0.95 mm^2^) as previously described [9]. The expression of VEGF-A, VEGFR1 (flt-1), and VEGFR2 (KDR/flk-1) was examined using immunohistochemistry with an anti-VEGF-A antibody (1:200, JH121, Merck Millipore, Tokyo, Japan), anti-VEGFR1 antibody (1:100, AF321, R&D Systems, Minneapolis, MN, USA), and anti-VEGFR2 antibody (1:600, 55B11, Cell Signaling Technology, Tokyo, Japan).

VEGF-A expression in the tumor cytoplasm or stroma was assessed as follows: ++, diffuse intense staining; +, diffuse faint staining; −, negative staining [9]. The expression of VEGFR1 and VEGFR2 on endothelial or tumor cell membrane/cytoplasm was assessed as the following: ++, staining in both vascular endothelial cells and tumor cells; +, staining only in vascular endothelial cells; −, negative staining [9].

The expression of HIF1α and CA9 was evaluated using immunohistochemistry with anti-HIF1α antibody (1:100, H-206, Santa Cruz Biotechnology, Dallas, TX, USA) and anti-CA9 antibody (1:50, H-120, Santa Cruz Biotechnology). The expression of HIF1α was assessed as the following: ++, expression in > 10% of tumor cells; +, expression in ≤ 10% of tumor cells; −, negative staining [9]. The expression of CA9 was assessed as the following: ++, universal strong expression around necrotic regions; +, occasional expression (typically around necrotic regions); −, negative staining [9].

The expression of nestin was examined using immunohistochemistry with an anti-nestin antibody (1:100, 10C2, Chemicon, Tokyo, Japan), and was assessed as a positive cell ratio analyzed in more than 1000 tumor cells from more than three areas, showing the representative appearance of each tumor [9].

### Statistical methods

One-way ANOVA with post-hoc analysis was used to compare MVD, and indices of MIB-1 and nestin-positive cell ratio. Analyses were performed with IBM SPSS statistics.
